# Subjective cognitive decline: opposite links to neurodegeneration across the Alzheimer’s continuum

**DOI:** 10.1093/braincomms/fcab199

**Published:** 2021-09-01

**Authors:** Elizabeth Kuhn, Audrey Perrotin, Clémence Tomadesso, Claire André, Siya Sherif, Alexandre Bejanin, Edelweiss Touron, Brigitte Landeau, Florence Mezenge, Denis Vivien, Vincent De La Sayette, Gaël Chételat, for the Alzheimer’s Disease Neuroimaging Initiative

**Affiliations:** Normandie Univ, UNICAEN, INSERM, U1237, PhIND “Physiopathology and Imaging of Neurological Disorders”, Institut Blood and Brain @ Caen-Normandie, Cyceron, 14000 Caen, France; Normandie Univ, UNICAEN, PSL Université, EPHE, INSERM, U1077, CHU de Caen, GIP Cyceron, NIMH, 14000 Caen, France; Normandie Univ, UNICAEN, INSERM, U1237, PhIND “Physiopathology and Imaging of Neurological Disorders”, Institut Blood and Brain @ Caen-Normandie, Cyceron, 14000 Caen, France; Normandie Univ, UNICAEN, PSL Université, EPHE, INSERM, U1077, CHU de Caen, GIP Cyceron, NIMH, 14000 Caen, France; Normandie Univ, UNICAEN, INSERM, U1237, PhIND “Physiopathology and Imaging of Neurological Disorders”, Institut Blood and Brain @ Caen-Normandie, Cyceron, 14000 Caen, France; Normandie Univ, UNICAEN, PSL Université, EPHE, INSERM, U1077, CHU de Caen, GIP Cyceron, NIMH, 14000 Caen, France; Normandie Univ, UNICAEN, INSERM, U1237, PhIND “Physiopathology and Imaging of Neurological Disorders”, Institut Blood and Brain @ Caen-Normandie, Cyceron, 14000 Caen, France; Normandie Univ, UNICAEN, INSERM, U1237, PhIND “Physiopathology and Imaging of Neurological Disorders”, Institut Blood and Brain @ Caen-Normandie, Cyceron, 14000 Caen, France; Normandie Univ, UNICAEN, INSERM, U1237, PhIND “Physiopathology and Imaging of Neurological Disorders”, Institut Blood and Brain @ Caen-Normandie, Cyceron, 14000 Caen, France; Normandie Univ, UNICAEN, INSERM, U1237, PhIND “Physiopathology and Imaging of Neurological Disorders”, Institut Blood and Brain @ Caen-Normandie, Cyceron, 14000 Caen, France; Normandie Univ, UNICAEN, INSERM, U1237, PhIND “Physiopathology and Imaging of Neurological Disorders”, Institut Blood and Brain @ Caen-Normandie, Cyceron, 14000 Caen, France; Normandie Univ, UNICAEN, INSERM, U1237, PhIND “Physiopathology and Imaging of Neurological Disorders”, Institut Blood and Brain @ Caen-Normandie, Cyceron, 14000 Caen, France; Département de Recherche Clinique, CHU Caen-Normandie, 14000 Caen, France; Normandie Univ, UNICAEN, PSL Université, EPHE, INSERM, U1077, CHU de Caen, GIP Cyceron, NIMH, 14000 Caen, France; Service de Neurologie, CHU de Caen, 14000 Caen, France; Normandie Univ, UNICAEN, INSERM, U1237, PhIND “Physiopathology and Imaging of Neurological Disorders”, Institut Blood and Brain @ Caen-Normandie, Cyceron, 14000 Caen, France

**Keywords:** subjective memory decline, memory awareness, anosognosia, neurodegeneration, clinical continuum of Alzheimer’s disease

## Abstract

Subjective memory decline is associated with neurodegeneration and increased risk of cognitive decline in participants with no or subjective cognitive impairment, while in patients with mild cognitive impairment or Alzheimer’s-type dementia, findings are inconsistent. Our aim was to provide a comprehensive overview of subjective memory decline changes, relative to objective memory performances, and of their relationships with neurodegeneration, across the clinical continuum of Alzheimer’s disease. Two hundred participants from the *Imagerie Multimodale de la maladie d'Alzheimer à un stade Précoce (IMAP+)* primary cohort and 731 participants from the Alzheimer’s Disease Neuroimaging Initiative (ADNI) replication cohort were included. They were divided into four clinical groups (*Imagerie Multimodale de la maladie d'Alzheimer à un stade Précoce*/Alzheimer’s Disease Neuroimaging Initiative): controls (*n* = 67/147, age: 60–84/60–90, female: 54/55%), patients with subjective cognitive decline (*n* = 30/84, age: 54–84/65–80, female: 44/63%), mild cognitive impairment (*n* = 50/369, age: 58–86/55–88, female: 45/44%) or Alzheimer’s-type dementia (*n* = 36/121, age: 51–86/61–90, female: 41/41%). Subjective and objective memory scores, and their difference (i.e. delta score reflecting memory awareness), were compared between groups. Then, voxelwise relationships between subjective memory decline and neuroimaging measures of neurodegeneration [atrophy (T1-MRI) and hypometabolism (^18^F-fluorodeoxyglucose-PET)] were assessed across clinical groups and the interactive effect of the level of cognitive impairment within the entire sample was assessed. Analyses were adjusted for age, sex and education, and repeated including only the amyloid-positive participants. In *Imagerie Multimodale de la maladie d'Alzheimer à un stade Précoce*, the level of subjective memory decline was higher in all patient groups (all *P* < 0.001) relative to controls, but similar between patient groups. In contrast, objective memory deficits progressively worsened from the subjective cognitive decline to the dementia group (all *P* < 0.001). Accordingly, the delta score showed a progressive decline in memory awareness across clinical groups (all *P* < 0.001). Voxelwise analyses revealed opposite relationships between the subjective memory decline score and neurodegeneration across the clinical continuum. In the earliest stages (i.e. patients with subjective cognitive decline or Mini Mental State Examination > 28), greater subjective memory decline was associated with increased neurodegeneration, while in later stages (i.e. patients with mild cognitive impairment, dementia or Mini Mental State Examination < 27) a lower score was related to more neurodegeneration. Similar findings were recovered in the Alzheimer’s Disease Neuroimaging Initiative replication cohort, with slight differences according to the clinical group, and in the amyloid-positive subsamples. Altogether, our findings suggest that the subjective memory decline score should be interpreted differently from normal cognition to dementia. Higher scores might reflect greater neurodegeneration in earliest stages, while in more advanced stages lower scores might reflect decreased memory awareness, i.e. more anosognosia associated with advanced neurodegeneration.

## Teaser

Kuhn et al. show that subjective memory decline should be interpreted differently across the continuum from normal cognition to Alzheimer’s dementia. In early stages, high subjective memory decline levels are linked to neurodegeneration, while in later stages those with lower subjective memory decline showed more brain alteration, likely reflecting anosognosia.

## Graphical Abstract

**Graphical Abstract:**
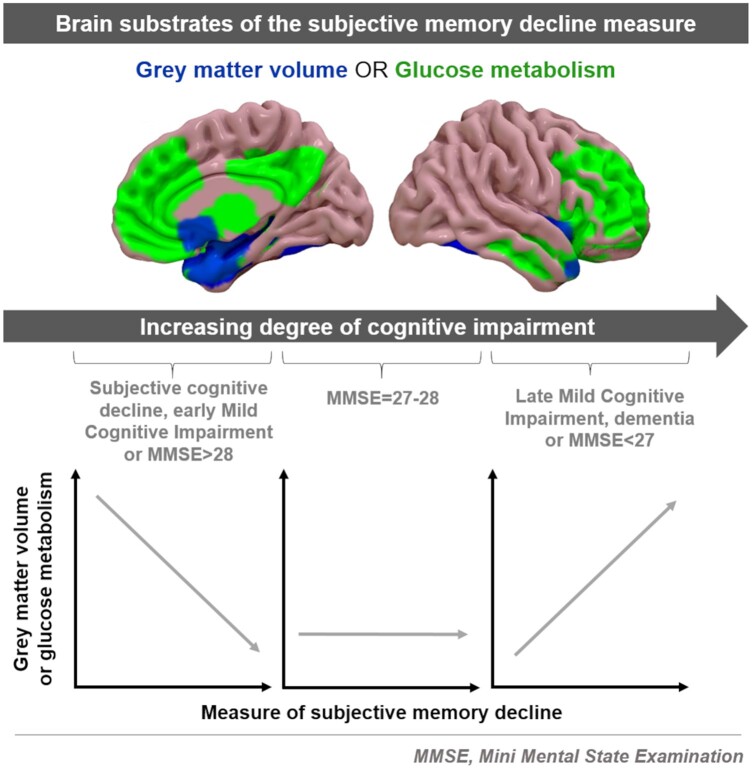

## Introduction

Subjective cognitive decline, also conceptualized as cognitive complaints, refers to the self-perception of worsening cognitive abilities relative to a previous level of performance.^1^ Such perception is often the warning signal that will motivate an individual to refer to a memory centre. Patients who experience a subjective decline in cognitive functioning but perform within the normal range for age, sex and education on objective cognitive tests are referred to as SCD patients.^1^ In SCD patients, the presence of subjective cognitive decline is known to be related to an increased risk of subsequent objective cognitive decline^2^^,^^3^ and conversion to dementia.^4^^,^^5^ Moreover, neuroimaging studies showed that SCD patients, when compared as a group to a control population, have increased levels of biomarkers suggestive of Alzheimer’s disease,^6^^,^^7^ including both amyloid deposition and neurodegeneration—i.e. hippocampal atrophy and/or temporoparietal hypometabolism.^8–12^ Altogether, data suggest that SCD patients are at greater risk for Alzheimer’s disease than cognitively unimpaired elderly without subjective cognitive decline, as their subjective cognitive decline would in part reflect subtle cognitive decline that has not yet reached the level of objective impairment required for the MCI diagnosis.^13^

In later stages, i.e. in patients with MCI or Alzheimer’s-type dementia, the meaning of subjective cognitive decline as regard to the potential underlying pathology is less clear. Few studies have assessed the cerebral substrates of subjective cognitive decline at these later stages, and most of them failed to show any association with amyloid deposition or grey matter volume.^14–16^ This might reflect the emergence of anosognosia corresponding to the progressive decrease in awareness of cognitive deficits. Anosognosia is indeed known to be present in the dementia, and even MCI, stages of Alzheimer’s disease.^17^^,^^18^ Thus, as cognitive awareness decreased, subjective cognitive decline may less accurately reflect cognitive impairment and the underlying neurodegeneration.

In the present study, we aim at providing a comprehensive overview of the changes in subjective cognitive decline and their neural substrates over the entire clinical continuum from normal cognition to Alzheimer’s-type dementia. As subjective decline in memory, rather than in other domains of cognition, increases the likelihood of Alzheimer’s disease in SCD patients,^1^^,^^4^^,^^19^ we used a score of SMD for this purpose. First, we compared the SMD score to a score of objective memory performances between clinical groups across the entire clinical spectrum from normal cognition to Alzheimer’s-type dementia. Second, we assessed the links between this SMD score and neurodegeneration (i.e. brain volume and glucose metabolism), both (i) within each clinical group and (ii) within the entire sample assessing the interactive effect of the level of cognitive impairment as measured with the MMSE. We repeated all analyses in two independent cohorts with complementary strengths.

## Materials and methods

### Participants

The analyses were performed on the *Imagerie Multimodale de la maladie d'Alzheimer à un stade Précoce* (IMAP+) primary cohort. Confirmatory analyses were run on an independent and complementary sample from the ADNI (www.adni-info.org). IMAP+ data were obtained at a single centre (Cyceron Center, Caen, France) with all participants scanned on the same MRI and PET cameras. ADNI data were multicentric and obtained from over 50 sites throughout the USA and Canada.

Two hundred participants from IMAP+ were included. There were 67 cognitively unimpaired elderly (controls), 36 SCD patients, 60 patients with MCI and 37 patients with dementia. Participants from the SCD, MCI and dementia groups were all recruited from local memory clinics. The full methodology for the cohort recruitment and evaluation was detailed in previous publications.^7^^,^^10^^,^^20^ Briefly, participants were all aged over 50 years, had at least 7 years of education, no history of alcoholism, drug abuse, head trauma, or psychiatric disorder. The inclusion and group classification of the participants were based on a clinical interview and a standardized neuropsychological assessment, according to internationally agreed criteria for SCD,^1^ MCI^21^ and probable Alzheimer’s disease^22^ with predominant amnestic deficits for as the so-called dementia group. As a first step, only clinical criteria were used to define these groups. Then as a second step, analyses were repeated including only amyloid-positive participants (see below). The IMAP+ study was approved by the local ethics committee (CPP Nord-Ouest III) and registered at http://clinicaltrials.gov (nb. NTC01638949). After a complete description of the study, written informed consent was obtained from all participants.

Data from 731 participants within ADNI were included for the replication study. There were 147 controls, 84 SCD, 369 MCI and 121 dementia patients. The full methodology for the cohort recruitment and evaluation can be found at http://adni.loni.usc.edu/. Briefly, controls and SCD patients had a global score of 0 at the CDR^23^ scale, MCI patients had a CDR score of 0.5 and patients with dementia had a CDR score of 1 or greater. In addition, MCI and dementia patients met standard diagnostic criteria for MCI^24^ or probable Alzheimer’s Disease,^22^ respectively. SCD patients from ADNI were not recruited from a memory clinic as in IMAP+; they were recruited from the general population and distinguished from the controls based on their level of subjective cognitive decline as reported by the participant himself, his/her partner, or the clinician, using the Cognitive Change Index (total score from first 12 items >16).^8^ The ADNI study was approved by the institutional review boards of all of the participating institutions. Informed written consent was obtained from all participants at each site.

### Neuropsychological assessments

In IMAP+ and ADNI, the neuropsychological assessment covered similar cognitive domains, although different tests were used. To aid comparability between cohorts and scores, SMD and objective memory raw scores described below were transformed into *w*-scores, i.e. age-, sex- and education-adjusted *z*-scores relative to controls.^25^^,^^26^

#### Subjective memory decline

In IMAP+, subjective cognitive decline was assessed with the CDS,^27^ a 39-item questionnaire that requires participants to rate how often they experience particular cognitive difficulties in everyday life on a 5-point scale (from ‘never’ = 0 to ‘very often’ = 4). Five items are usually removed as they are too strongly related to sex and age-specific cultural norms (e.g. related to cooking or sewing),^28^ leading to the so-called reduced-CDS score corresponding to the sum of the 34 remaining items.^27^ Because subjective decline in memory is more likely to be associated with Alzheimer’s disease than other domains of cognition (see ‘Introduction’ section), we used a memory score derived from the CDS as defined in a previous publication based on a factorial analysis.^29^ The memory score was found to be the most sensitive to the evolution of the disease as it best discriminated SCD patients from both MCI and controls.^29^ The memory score was thus used as the SMD score for the following analyses from the IMAP+ cohort.

In ADNI, subjective cognitive decline was assessed with the ECog,^30^ a 39-item questionnaire that requires participants to rate the ability to perform everyday tasks now as compared to the ability to do these same tasks 10 years earlier on a 4-point scale (from ‘no change’ = 1 to ‘consistently worse’ = 4). As for IMAP+, we used a memory subscore derived from a factorial analysis conducted on this scale^30^ as the SMD score.

The findings presented in the core article were obtained using these SMD scores. For the sake of completeness, analyses were repeated using a score of global subjective cognitive decline (i.e. the reduced-CDS score for IMAP+ and the total ECog score for ADNI), and the corresponding findings were reported as Supplementary data.

#### Objective cognitive performances

In IMAP+, objective episodic memory performances were assessed using the delayed recognition subscores of the ESR task (mean of the subscores from the two 16-word lists).^31^ In ADNI, objective episodic memory performances were assessed using the Immediate subscore of the RAVLT (sum of the five trials of the 15-word list).^32^ In both cohorts, objective global cognition was assessed using the MMSE.^33^ Note that higher scores indicated better objective memory/cognitive performances for all tests.

#### Index of memory awareness (delta score)

In IMAP+ and ADNI, an index of memory awareness, called as the delta score, was computed for each participant.^34^^,^^35^ The delta score was obtained by subtracting the reversed SMD *w*-score from the objective memory *w*-score. More specifically, (i) the two scores of interest were standardized and transformed into age-, sex- and education-adjusted *z*-scores relative to controls, corresponding to w-scores; (ii) the SMD score was reversed so that, as for the objective memory score, a high score indicated a high self-rated level of performance; (iii) then the reversed SMD *w*-score was subtracted from the objective memory *w*-score. A positive delta score indicated that the participant overestimated his/her difficulties as compared to his/her objective performances, while a negative delta score indicated that the participant underestimated his/her difficulties, likely reflecting anosognosia.^36^

### Neuroimaging acquisition and processing

In IMAP+, high-resolution T_1_-weighted anatomical MRI, FDG-PET and ^18^F-Florbetapir-PET images were acquired to measure grey matter volume, cerebral glucose metabolism and amyloid deposition, respectively. All participants were scanned at the Cyceron Center (Caen, France), on the same MRI (Philips Achieva 3.0 T scanner) and PET (Discovery RX VCT 64 PET-CT) cameras. The detailed acquisition and pre-processing procedure is available in previous publications,^37^^,^^38^ and provided in the Supplementary material 1. Briefly, MRI images were segmented and normalized to the Montreal Neurological Institute space, PET images were preprocessed using MRI for co-registration and normalization, and participant uptake values were extracted in a predetermined neocortical mask on Florbetapir-PET data. Participants were classified as amyloid (florbetapir) positive or negative using a threshold based on healthy young controls (see Supplementary material 1).

For ADNI, full information regarding the acquisition of anatomical MRI, FDG- and Florbetapir-PET data is provided at http://adni.loni.usc.edu/data-samples/data-types/. ADNI participants were selected to have a maximum interval of 3 months between the three neuroimaging examinations. To allow comparison between cohorts, IMAP+ pre-processing procedures were replicated on ADNI data.

For the sake of completeness, all analyses with FDG-PET were repeated using PVC images to ensure that the results did not reflect partial volume effects. Moreover, all analyses were repeated in a subgroup only including amyloid-positive participants to assess whether results remain the same in participants within the Alzheimer’s continuum. The sample size for each corresponding analysis is indicated in Supplementary Table 1.

### Statistical analysis

Differences in demographic and clinical variables were assessed both across clinical groups within each cohort, and across cohorts within each clinical group, using ANOVA and *post hoc* Tukey-tests for continuous variables, and using χ^2^ tests for categorical variables.

To compare the SMD, objective memory and delta scores between clinical groups, ANOVA were performed. The percentage of participants with a positive/negative delta score was also compared between clinical groups using χ^2^ tests.

To determine the relationships between the SMD score and neurodegeneration (i.e. atrophy and glucose hypometabolism) within each clinical group, a voxelwise full factorial design was carried out using the SPM12. In brain regions where a statistically significant correlation was found, values were extracted in order to plot the correlation between these neuroimaging values and the SMD score using the R software. Then, a voxelwise full factorial design was carried out in SPM12 in order to test the interactive effect of the level of cognitive impairment, as measured by the MMSE score, on the link between the SMD score and neurodegeneration – including atrophy or glucose hypometabolism. In brain regions where there was a statistically significant interaction, neuroimaging values were extracted in order to plot the correlation between these neuroimaging values and the SMD score for each MMSE tertile score using the R software. The influence of age, sex and education was regressed out in all statistical models. For the sake of completeness, the interactive effect of the level of cognitive impairment was also tested using a score of episodic memory (i.e. the ESR or RAVLT according to the cohort) instead of the MMSE.

All statistical analyses were performed in both independent cohorts (i.e. IMAP+ primary cohort and ADNI replication cohort). Statistical analyses were performed with statistical significance set at *P* < 0.05, using R (version 3.6.1; https://cran.r-project.org/bin/windows/base/old/3.6.1/). For neuroimaging analyses, the statistical significance was set at *P*_uncorrected_<0.005 combined with a minimum cluster size determined by Monte-Carlo simulation using the Cluster-Sim program to achieve a statistical significance corrected for multiple comparisons of *P* < 0.05 (Supplementary Table 2). Results obtained at the same statistical threshold but with a lower cluster size were considered as trends.

### Data availability

IMAP+ data used within this study are not publicly available due to them containing information that could compromise participant consent and confidentiality but they are available from the corresponding author to research groups wishing to reproduce/confirm results under reasonable request, and pending approval by the study coordinator. ADNI data used in this study were obtained from the public ADNI database (adni.loni.usc.edu).

## Results

### Descriptive statistics

Demographic and clinical characteristics of the study participants within each cohort, as well as differences between-groups and between cohorts, are indicated in Table 1. Within cohorts, expected between-groups differences were observed regarding global cognition, the proportion of Apolipoprotein E allele ε4 and amyloid SUVr. Between-cohort comparisons showed that IMAP+ participants were younger (*P* < 0.001 for all groups except the MCI, *P* = 0.67) and less educated (*P* < 0.001 for all groups) than ADNI participants. Moreover, amyloid SUVr was lower in IMAP+ than in ADNI for controls (*P* < 0.001) and SCD patients (*P* = 0.01), and the MMSE score was lower in IMAP+ than in ADNI for MCI and dementia patients (all *P* < 0.001; Table 1).

**Table 1.** Demographic and clinical characteristics of participants from the two independent cohorts

	Controls	SCD	MCI	Dementia	*P* **-value (*F*-value)**	*Post hoc* Tukey-test, pairwise comparisons
**IMAP+ primary cohort (*n* = 200)**						
*n*	67	36	60	37		
Sex: female % (*n*)	53.73 (36)	44.44 (16)	45.00 (27)	40.54 (15)	0.57^a^ (2.03)	
Age	70.10 ± 6.34	67.47 ± 7.51	72.28 ± 7.35	68.38 ± 10.05	**0.01** ^b^ (3.66)	MCI>SCD, dementia (*P* = 0.02, *P* = 0.07)
Education	12.33 ± 3.76	13.22 ± 3.59	11.48 ± 3.60	10.84 ± 3.10	**0.02** ^b^ (3.31)	SCD>MCI, dementia (*P* = 0.1, *P* = 0.02)
Global cognition: MMSE	28.95 ± 1.04	29.00 ± 1.06	26.92 ± 1.80	20.43 ± 4.61	**<0.001** ^b^ (118.96)	Controls, SCD>MCI>dementia (all *P* < 0.001)
APOEε4: carrier % (*n*)	23.44 (15)	13.79 (4)	48.08 (25)	72.73 (24)	**<0.001** ^a^ (32.04)	Controls, SCD<MCI<dementia (all *P* < 0.05)
Amyloid SUVr	1.20 ± 0.13	1.22 ± 0.12	1.39 ± 0.24	1.60 ± 0.26	**<0.001** ^b^ (32.38)	Controls, SCD<MCI<dementia (all *P* < 0.005)
**ADNI replication cohort (** *n* ** = 731)**						
*n*	157	84	369	121		
Sex: female % (*n*)	54.77 (86)	63.10 (53)	44.44 (164)	40.50 (49)	**0.002** ^a^ (15.15)	Controls>MCI, dementia (*P* = 0.04, *P* = 0.03) SCD>MCI, dementia (*P* = 0.003, *P* = 0.002)
Age	**74.21 ± 5.95** ^c^	**72.00 ± 5.41** ^c^	71.84 ± 7.40	**74.29 ± 7.75** ^c^	**<0.001** ^b^ (6.67)	SCD<controls, dementia (both *P* = 0.09); MCI<controls, dementia (*P* = 0.002, *P* = 0.004)
Education	**16.57 ± 2.40** ^c^	**16.79 ± 2.54** ^c^	**16.27 ± 2.64** ^c^	**15.95 ± 2.61** ^c^	0.08^b^ (2.27)	SCD> dementia (*P* = 0.1)
Global cognition: MMSE	29.00 ± 1.22	28.98 ± 1.13	**28.07 ± 1.73** ^c^	**23.08 ± 2.14** ^c^	**<0.001** ^b^ (368.35)	Controls, SCD>MCI> dementia (all *P* < 0.001)
APOEε4: carrier % (*n*)	25.48 (40)	29.76 (25)	49.59 (183)	65.29 (79)	**<0.001** ^a^ (56.28)	Controls<MCI, dementia (both *P* < 0.001); SCD<MCI<dementia (all *P* < 0.005)
Amyloid SUVr	**1.32 ± 0.23** ^c^	**1.35 ± 0.22** ^c^	1.44 ± 0.32	1.60 ± 0.25	**<0.001** ^b^ (25.61)	Controls, SCD<MCI<dementia (*P* < 0.001, except SCD<MCI *P* = 0.07)

Values are expressed as mean ± 1SD or percentage (sample size). *P* < 0.05 in bold.

APOEε4, allele ε4 of Apolipoprotein E; *n*, sample size; SD, standardized deviation.

a χ^2^ between clinical groups (i.e. controls, SCD, MCI, dementia).

b ANOVA between clinical groups (i.e. controls, SCD, MCI, dementia), *post hoc* Tukey pairwise tests.

c ANOVA between cohorts: IMAP+ versus ADNI for each clinical group (i.e. controls, SCD, MCI, dementia).

### Subjective memory decline, objective memory and delta scores across clinical groups

**Figure 1:**
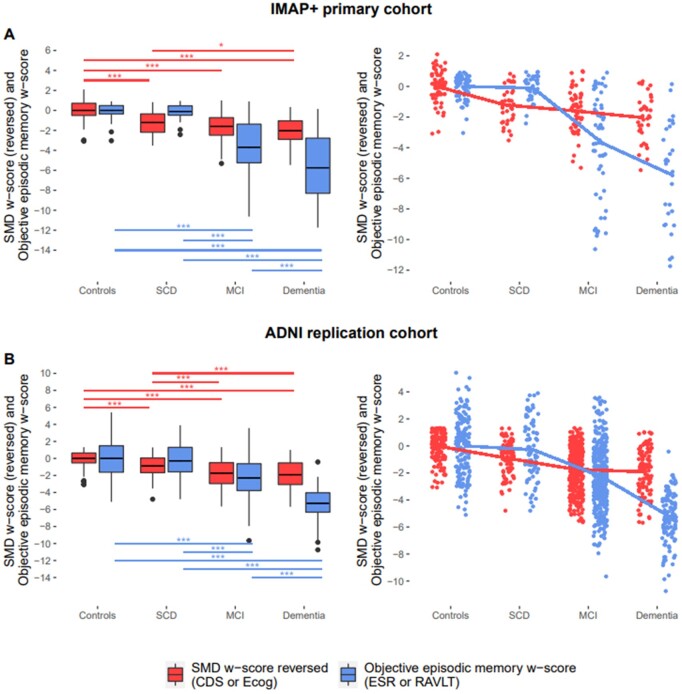
**Between-group comparisons of the mean subjective and objective episodic memory scores within the two independent cohorts. Scores correspond to reversed SMD *w*-scores (in red) and objective episodic memory *w*-scores (in blue), such that lower scores indicate greater subjective memory decline and poorer memory performances, respectively**. (**Left panel**) Boxplot illustrating the results of *post hoc* Tukey-test after ANOVA showing the mean (bold horizontal line), interquartile range (box), total range (whiskers) and outliers (black dots). (**Right panel**) Graphs indicating the distribution of participants’ values and average for each clinical group connected by a line. (**A**) Data for the IMAP+ primary cohort, with the CDS in red and the ESR in blue. (**B**) Data from the ADNI replication cohort, with the Ecog in red and the RAVLT in blue. *P < 0.05, **P < 0.01, ***P < 0.001 for the between-group comparisons.

**Figure 2:**
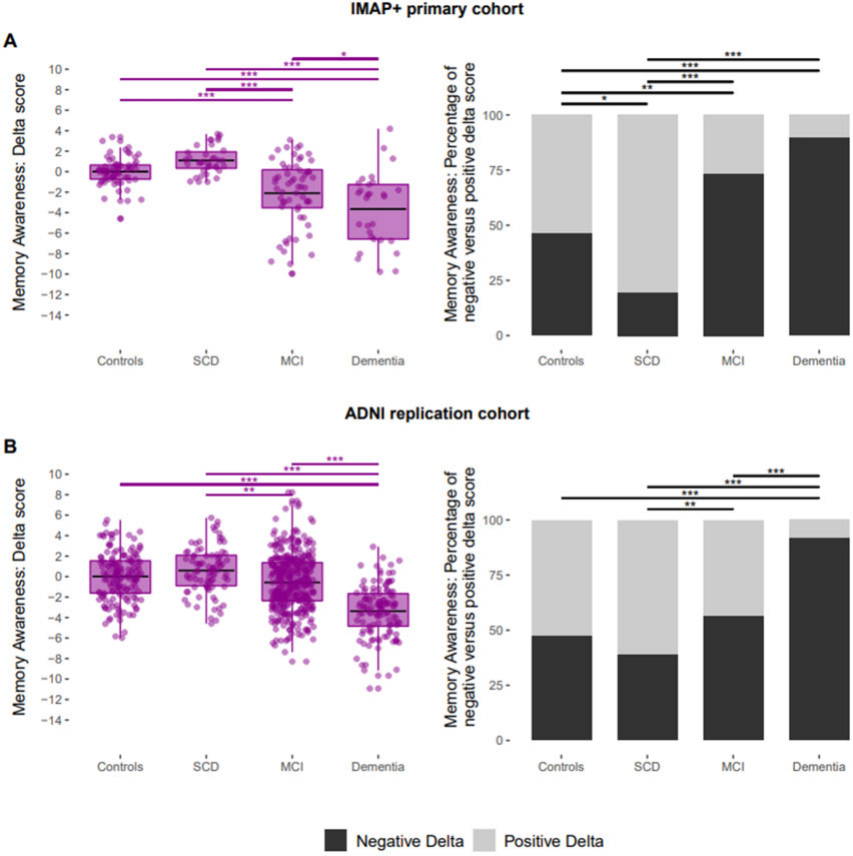
**Clinical group comparisons of the mean delta score within the two independent cohorts. Scores correspond to delta scores (in purple), i.e. the subtraction of the reversed SMD from the objective episodic memory *w*-score**. (**Left panel**) Graphs indicate boxplot and results of *post hoc* Tukey-test after ANOVA showing the mean (bold horizontal line), interquartile range (box), total range (whiskers) and outliers (black dots). (**Right panel**) Histograms illustrating the percentage of negative versus positive delta scores for each clinical group and results of χ^2^ tests of the pairwise between-group comparisons of these percentages. Positive delta scores indicate that participants overestimate their difficulties as compared to their objective performance, while negative delta scores indicate that participants underestimate their difficulties, likely reflecting anosognosia. (**A**) Data for the IMAP+ primary cohort. (**B**) Data for the ADNI replication cohort. *P < 0.05, **P < 0.01, ***P < 0.001 for the between-group comparisons.

In IMAP+, between-group comparisons showed that the group mean SMD score was higher in all patient groups versus controls, but did not significantly differ between patient groups, except between SCD and dementia patients. The mean objective memory score significantly decreased from controls and SCD patients to MCI to dementia patients (Fig. 1A). The mean delta score progressively decreased from controls and SCD patients to MCI to dementia patients (Fig. 2A left side). Moreover, the percentage of participants with a negative delta score significantly decreased from controls (46.27%) to SCD patients (19.44%) and increased from controls and SCD patients to patients with MCI (73.33%) and dementia (89.66%) (Fig. 2A right side).

In ADNI, between-group comparisons showed that the group mean SMD score significantly increased from controls to SCD patients to patients with MCI and dementia; whereas the mean objective memory score significantly decreased from controls and SCD patients to MCI to dementia patients (Fig. 1B). The mean delta score decreased in patients with dementia compared to the three other groups and in MCI compared to SCD patients (Fig. 2B left). Moreover, the percentage of participants with a negative delta score significantly increased from controls (47.77%) to dementia patients (91.73%) and from SCD (39.29%) to MCI to dementia patients, while it tended to increase from controls to MCI patients (56.79%, *P* = 0.07) (Fig. 2B right).

### Relationships between subjective memory decline and neurodegeneration

#### Voxelwise multiple regressions with FDG-PET and MRI across clinical groups

**Figure 3:**
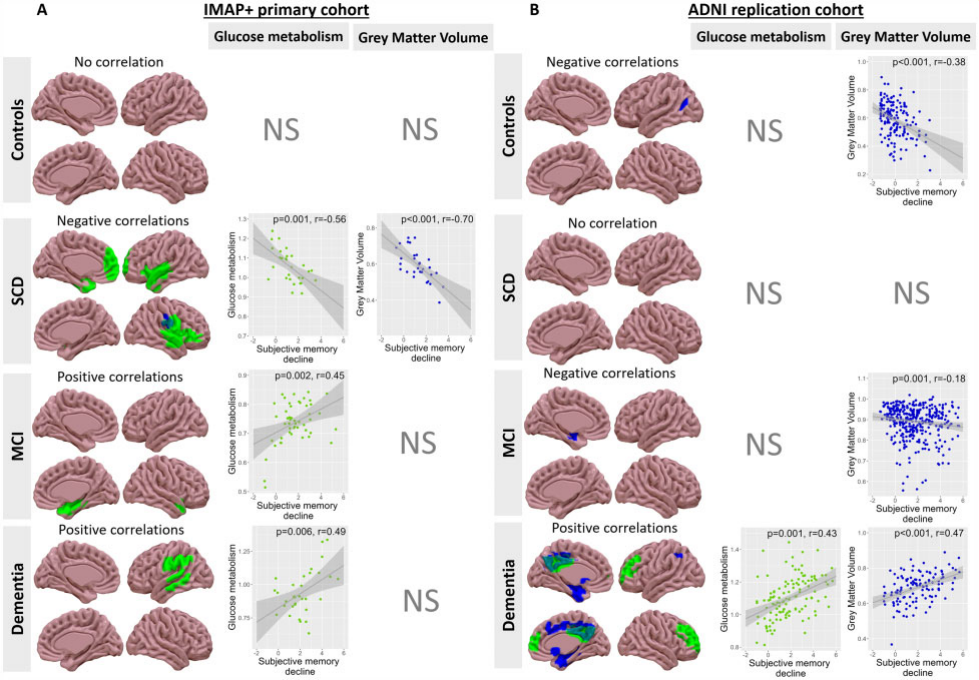
**Relationships between subjective memory decline and neurodegeneration through voxelwise multiple regressions in each clinical group from the two independent cohorts. Brain representations show the results of the voxelwise correlations between the SMD score and either glucose metabolism (green) or grey matter volume (blue) thresholded at *P* < 0.005 combined with a cluster-level correction for multiple comparisons; and graphs on the right side illustrate the corresponding regressions. All analyses were adjusted for age, sex and education**. (**A**) Data for the IMAP+ primary cohort. (**B**) Data for the ADNI replication cohort. NS, Not Significant.

In IMAP+, voxelwise multiple regressions revealed significant associations between the SMD score and glucose metabolism within each patient group, but in opposite directions according to the clinical group. In SCD patients, a higher SMD score correlated with lower glucose metabolism in the temporal and frontal cortex, insula and putamen; while in patients with MCI or with dementia, a lower SMD score correlated with lower glucose metabolism in the medial temporal lobe and the temporo-parietal region, respectively. The SMD score was also significantly associated with grey matter volume in SCD patients, where a higher SMD score correlated with lower grey matter volume in the supramarginal gyrus. No association was found in the controls (Fig. 3A).

In ADNI, similar opposite relationships were observed with a trend for negative correlations in early stages and positive correlations in late stages. However, there were differences in the specific clinical groups where the relationship was significant, and the topography of the results also differed. Thus, in controls, a higher SMD score was related to lower grey matter volume in the angular gyrus (Fig. 3B), while in dementia patients a lower SMD score was associated with lower glucose metabolism and grey matter volume in the posterior cingulate cortex, precuneus and frontal areas, as well as lower grey matter volume in the medial temporal lobe, hypothalamus and nucleus accumbens. The MCI showed an intermediate profile with a slighter (*r* = 0.18 compared to 0.38 in controls) but significant negative relationships such that a higher SMD score was associated with lower grey matter volume in the hippocampus and amygdala.

#### Interactive effect of the level of cognitive impairment (MMSE)

**Figure 4:**
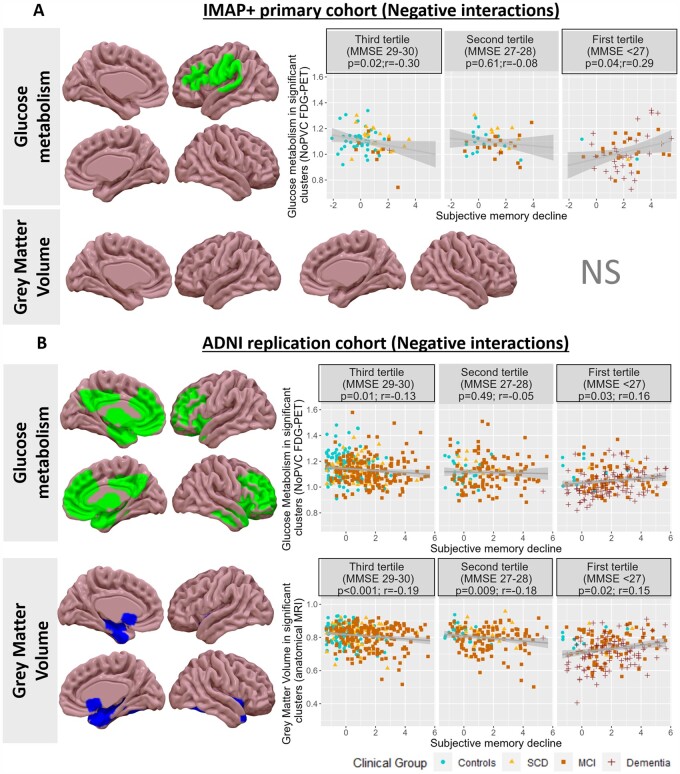
**Negative interactive effect of the level of cognitive impairment (MMSE) on the voxelwise relationships between the SMD score and neurodegeneration within the two independent cohorts. Brain representations** (**left panel**) show the results of the voxelwise negative interactions between the SMD and the MMSE scores on glucose metabolism (green) or grey matter volume (blue) thresholded at *P* < 0.005 combined with a cluster-level corrected for multiple comparisons; and graphs (right panel) illustrate the regression in the corresponding brain areas for each MMSE tertile. All analyses were adjusted for age, sex and education. (**A**) Data for the IMAP+ primary cohort. (**B**) Data for the ADNI replication cohort. NS, Not Significant.

In IMAP+, a negative interaction was found between the MMSE and the SMD scores on glucose metabolism in temporo-parietal regions. The interactive effect was such that a higher SMD score was related to lower metabolism in participants in the highest MMSE tertile (i.e. >28), while a lower SMD score was related to lower metabolism in participants in the lowest MMSE tertile (i.e. <27). No significant correlation was found in participants in the intermediate MMSE tertile (Fig. 4A).

In ADNI, results were globally recovered for glucose metabolism with a negative interaction between SMD and MMSE scores, although in partly distinct brain regions, i.e. in the medial temporal lobe, posterior cingulate cortex, precuneus, prefrontal and anterior cingulate cortex, insula, caudate nucleus and thalamus. In contrast to what was observed on IMAP+ data, a negative interaction between SMD and MMSE scores was also found on grey matter volume in the medial temporal lobe and caudate nucleus. The interactive effect was such that a higher SMD score was related to lower metabolism and lower grey matter volume in participants in the highest MMSE tertile (i.e. >28), while a lower SMD score was related to lower metabolism and grey matter volume in participants in the lowest MMSE tertile (i.e. <27). In the intermediate MMSE tertile, a higher SMD score was only related to lower grey matter volume (Fig. 4B).

There was also a positive interaction in the IMAP+ cohort between the MMSE and the SMD scores on grey matter volume in the occipital cortex showing a link in participants in the highest MMSE tertile (i.e. >28) only, while no significant association was found in the other MMSE tertile groups. Yet, this positive interaction was not recovered in the ADNI replication cohort and no other positive interaction was found.

### Additional analyses

A series of additional analyses were conducted for confirmation purposes.

#### Interactive effect of the level of memory impairment

The analyses assessing the interactive effects of the level of cognitive impairment on the relationships between the SMD score and neurodegeneration were repeated using a measure of episodic memory (the ESR or RAVLT *w*-scores depending on the cohort) instead of the MMSE score. Results were similar to those obtained with the MMSE, showing a negative interaction between the episodic memory score and the SMD score on glucose metabolism, in both IMAP+ and ADNI cohorts, in extended brain areas including frontal, temporal and temporo-parietal areas as well as the posterior cingulate and precuneus. No such interactive effect was detected on grey matter volume, however, in any of the two cohorts (Supplementary Fig. 1).

#### Relationships between subjective memory decline and neurodegeneration using PVC images

Neuroimaging analyses were replicated using PVC FDG-PET images. Findings were very similar with only slight differences in the level of significance or size of clusters as illustrated in Supplementary Fig. 2 and Supplementary Fig. 3.

#### Links between subjective memory decline and neurodegeneration in amyloid-positive participants

For both cohorts, all neuroimaging analyses were repeated including only the amyloid-positive participants and results were overall comparable. Indeed, the opposite relationship between the SMD score and glucose metabolism or grey matter volume across the Alzheimer’s continuum was recovered (i.e. negative correlations in controls, SCD and/or MCI patients; while positive correlations in MCI and/or dementia patients according to the cohort), together with the negative interactive effect of the level of cognitive or memory impairment on these voxelwise relationships (Supplementary Fig. 4 and Supplementary Fig. 5). Subtle differences were yet observed in the statistics such that some of these relationships did not survive to the cluster-level correction for multiple comparisons (likely due to the reduction in sample sizes), while some relationships became significant (e.g. the negative correlation between the SMD score and glucose metabolism in the IMAP+ controls) but the overall pattern remained the same.

#### Relationships between subjective cognitive decline and neurodegeneration

All analyses were finally repeated using a measure of subjective cognitive decline (the reduced-CDS or total-Ecog depending on the cohort) instead of subjective memory decline (the SMD score). Findings were very similar with only slight differences in the level of significance or the size of clusters as illustrated in Supplementary Fig. 6 and Supplementary Fig. 7.

## Discussion

The current study aimed at providing a comprehensive overview of subjective memory decline across the clinical continuum from cognitively unimpaired elderly to patients with Alzheimer’s-type dementia. Our main findings were that (i) the gap between subjective memory decline and objective memory performances increased across clinical stages, with a decrease in memory awareness at the dementia stage, which could start from the MCI stage; and (ii) the pattern of association between subjective memory decline and neurodegeneration changed across clinical groups and according to the level of cognitive impairment. A negative association was found in participants at early stages of the clinical continuum (i.e. in SCD/MCI patients in IMAP+/ADNI, or in participants with an MMSE score >28 in both cohorts), where a higher SMD score correlated with lower grey matter volume and/or glucose metabolism. In contrast, a positive association was found at later stages (i.e. in patients with MCI and/or dementia, or in participants with an MMSE score <27), where a lower SMD score was related to greater neurodegeneration.

### Memory awareness decreased from the MCI stage

This study showed that the SMD score was higher in all patient groups compared to controls, but only marginally increased from the SCD stage (significantly only from SCD to dementia in IMAP+, and significantly from SCD to MCI, but not from MCI to dementia, in ADNI). In contrast, as expected, the objective memory score was not altered in SCD compared to controls, but declined from SCD to MCI and again from MCI to dementia stages. The extent of the difference between the subjective (SMD) and the objective memory scores within each clinical group has been specifically assessed with the delta score, an index of memory awareness. The results indicated that the level of memory awareness in SCD patients did not differ from controls, although the percentage of participants with a positive delta score was higher in SCD patients compared to controls (only significant in the IMAP+ cohort). SCD patients, especially those recruited from memory clinics, thus tended to overestimate their memory difficulties relative to their performance in objective tests, which was expected as SCD patients are defined as cognitively unimpaired elderly who subjectively experience a decline in cognitive functioning. These results might indicate that SCD patients perceive subtle changes in their memory functioning that cannot (yet) be objectivized by the standard memory tests which may lack sensitivity. By contrast, patients with MCI and/or dementia both showed a lower level of memory awareness, with a higher percentage of participants with a negative delta score, compared to controls and/or SCD patients, with MCI patients being intermediate between controls/SCD and dementia patients. Later clinical stages are thus characterized by a lower memory awareness suggestive of anosognosia, classically described in patients with dementia and thought to start as early as the MCI stage.^39^ This is in line with previous studies that indicated a frequency of anosognosia symptoms increasing across the clinical continuum of Alzheimer’s disease and over time.^17^^,^^40–42^

### Opposite links between subjective memory decline and neurodegeneration across the clinical continuum

The increasing level of anosognosia across the clinical stages of Alzheimer’s disease might explain the switch we found in the relationships between the SMD score and neurodegeneration across clinical groups or depending on the level of cognitive impairment. Specifically, in the less impaired participants, a higher SMD score was related to less glucose metabolism; while at later stages, in participants with objective cognitive impairment, a lower SMD score was associated with less glucose metabolism. Interestingly, this general trend was found in both cohorts. In ADNI, these findings were extended to grey matter volume, likely reflecting the higher statistical power associated with increased sample sizes. There were yet differences between cohorts in the specific clinical group where these relationships were significant. For instance, the negative relationship between the SMD score and neurodegeneration neuroimaging biomarkers found in the IMAP+ SCD patients was not recovered in the ADNI SCD but was found in the ADNI MCI patients instead. These differences probably reflected the differences in the definitions of the clinical groups. Thus, the ADNI SCD patients were not recruited from memory clinics as in IMAP+ but from the community, and they might be closer to the IMAP+ controls. Consistently, the percentage of SCD patients with a positive delta score was higher than that in controls in IMAP+ while it was the same as in controls in ADNI. As for the MCI patients, they also seem to be less advanced in the clinical continuum in ADNI than in IMAP+, as indicated by their higher MMSE score, making them cognitively close to the IMAP+ SCD patients. Consistently, MCI patients did not significantly differed from controls in terms of memory awareness in ADNI (delta score and percentage of negative delta), while they had a significantly lower memory awareness in IMAP+. The switch in the relationships between the SMD score and neurodegeneration therefore existed in both cohorts but was highlighted between different clinical groups. A negative relationship was also found between the SMD score and neurodegeneration in the ADNI controls; given the location of this cluster (in the very bottom of a lateral occipital sulcus), this result is thought to reflect spurious effect due to inter-individual variability in the anatomy of this region. Interestingly, when using the interactive approach with the level of cognitive impairment instead of clinical groups, these differences were annihilated and the same general effects could be recovered in both cohorts. Although correlation coefficients were relatively weak, negative links between SMD score and glucose metabolism were found in participants with MMSE >28, whereas there were positive links in participants with MMSE <27. The topography yet partly differed between cohorts, which could also reflect overall differences in the cohorts (e.g. differences in the amyloid SUVr and in the education level), although no direct relationships were found between education level and the SMD or delta scores (see Supplementary Table 3).

Our findings are overall consistent with previous studies showing that, in SCD patients, higher SMD score was related to greater neurodegeneration.^6^^,^^7^ Brain regions found to be involved are also similar to those highlighted in previous studies, including the (para-)hippocampal area for grey matter volume^8^^,^^10^ and prefrontal and temporal cortex for glucose metabolism.^12^^,^^43^^,^^44^ The insula was not reported in previous studies, but its involvement, as highlighted here, might reflect the role of this structure in the salience network^45^ and interoception/metacognitive processes.^46^^,^^47^ Finally, brain regions related to the SMD score in SCD patients also partly overlap with AD-sensitive areas (in the hippocampus and temporo-parietal cortex), although not encompassing more posterior associative brain regions. Altogether, this suggests that, at this stage (i.e. in SCD patients recruited from memory clinics), a high SMD score might reflect different processes including normal ageing, sleep disorders, anxiety and depression, and possibly as well Alzheimer’s disease-related neurodegenerative processes.^48^

At later stages, i.e. in patients with MCI and/or dementia, the relationships found between the SMD score and both grey matter volume and glucose metabolism were in the opposite direction, with a lower score being associated with lower grey matter volume and glucose metabolism. Only a few studies investigated the neuroimaging correlates of the SMD score, and they did not find an association with grey matter volume,^14–16^ and only one used FDG-PET and found lower SMD score linked to lower glucose metabolism.^49^ Several studies however assessed the links with anosognosia at these stages, corresponding to the discrepancy between SMD and objective memory scores or between self- and informant-report of memory decline. These studies showed a link between anosognosia and reduced glucose metabolism in frontal^49–51^ and temporo-parietal^35^^,^^52–54^ regions, or with reduced volume in the prefrontal and cingulate cortex,^54^^,^^55^ and in the medial temporal lobe.^56^ In the present study, the brain regions found to be significantly associated with the SMD score across cohorts and groups included the precuneus, posterior cingulate, prefrontal, medial temporal cortex and temporo-parietal regions. These brain regions are overall very similar to the brain regions found to be associated with anosognosia in previous studies, suggesting that a lower SMD score could reflect the onset of a decreased awareness in MCI and dementia patients. Moreover, these regions are part of the Default Mode Network – thought to be involved in self-related processes and memory – and include the brain regions the most sensitive to AD.^57^^,^^58^ Hence, in later stages of the disease, a lower SMD score seems to be reflective of higher anosognosia and more advanced AD-related neurodegeneration, consistent with previous studies showing that higher anosognosia was linked to worse disease burden profile.^59–61^

### Strength and limitations

The major strength of the current study was to provide an overview of the changes in subjective memory decline and its links with neurodegeneration across the entire clinical continuum from normal cognition to Alzheimer’s-type dementia. Most of previous studies have focused on one or another stage of this continuum, and this overall assessment allowed us to get a more comprehensive understanding of the differential meaning of this measure depending on the clinical stage. In addition, we repeated our analyses in two independent cohorts (i.e. IMAP+ and ADNI), and using different methodological approaches (i.e. across clinical groups versus according to the level of cognitive or memory impairment), and were able to recover the switch in the relationships between the SMD score and neurodegeneration across clinical groups or depending on the level of cognitive impairment. We also ensured that the results with FDG-PET were not significantly impacted by partial volume effects, and repeated all analyses in a subsample of amyloid-positive participants. Our findings thus appear to be reliable as they were overall recovered in supplementary analyses.

However, some limitations must be noted. First, mean differences between groups were reported with yet relatively large within-group variability in the measures so that effect sizes were relatively small; this variability might lead to detect spurious relationships – as also mentioned above for the results in the ADNI controls. Second and as discussed above, the definitions of the clinical groups differed according to the cohort and led to some differences in the results. Third, the results are not representative of the general population as most participants were white, Caucasian and highly educated; further studies on more diverse populations are needed. Fourth, the study was cross-sectional, so the links with clinical or neurodegeneration changes over time were not assessed. Moreover, the study focused on subjective memory decline (or a global score of subjective cognitive decline) but did not assess the links with the subjective decline in other cognitive domains. Future studies are needed to further assess the changes, e.g. in language or executive functions, across the clinical continuum of Alzheimer’s disease and their links with neurodegeneration, especially as their association with future cognitive decline or dementia is poorly understood.^62^^,^^63^ It is also important to note that there are currently no validated measures of subjective cognitive or memory decline, ranging from a simple question to more or less comprehensive scales.^63^^,^^64^ Here, the scales used in both IMAP+ and ADNI cohorts were based on several items, and it would be interesting to determine which items from these scales are the most relevant for a better clinical use of these measures. Such questions are actually addressed in projects related to the SCD-initiative.^1^ Additionally, there was a growing interest for the informant-reported cognitive decline, and it would be interesting to determine if the patterns found with the SMD score would be similar or different across the clinical continuum of Alzheimer’s disease using the informant report.

## Conclusion

As a whole, our findings suggest that subjective memory decline should be interpreted differently across the clinical continuum from normal cognition to Alzheimer’s-type dementia, with an opposite association between SMD score and neurodegeneration according to the clinical group or the level of cognitive impairment. At early stages – in SCD patients recruited from memory clinics and participants with MMSE >28 – a higher SMD score was linked to greater neurodegeneration and likely indicates an increased risk of Alzheimer’s disease. Conversely, at later stages – in patients with MCI and/or dementia or in participants with MMSE <27 – participants underestimate their difficulties as they tend to show anosognosia, such that their lower SMD score was linked to greater neurodegeneration and would rather reflect more advanced disease progression.

## Supplementary material

Supplementary material is available at *Brain Communications* online.

## Supplementary Material

fcab199_Supplementary_Data

## Abbreviations

ADNI =: Alzheimer’s disease neuroimaging initiative
CDR =: Global Clinical Dementia Rating Scale
CDS =: Cognitive Difficulties Scale
ECog =: Everyday Cognition Questionnaire
ESR =: encoding, storage and retrieval
FDG =: ^18^F-fluorodeoxyglucose
IMAP+ =: Imagerie Multimodale de la maladie d'Alzheimer à un stade Précoce
MCI =: patients with mild cognitive impairment
MMSE =: Mini Mental State Examination
NoPVC =: partial volume effects non-corrected
PVC =: partial volume effects-corrected
RAVLT =: Rey Auditory Verbal Learning Test
SCD =: patients with subjective cognitive decline
SMD =: subjective memory decline
SPM12 =: Statistical Parametric Mapping software
SUVr =: global neocortical standardized uptake value ratio
