# Supplementary material for: Subjective cognitive decline: opposite links to neurodegeneration across the Alzheimer’s continuum
Source: Brain Commun. 2021 Sep 1;3(3):fcab199. doi: 10.1093/braincomms/fcab199 (PMC8421692; doi:10.1093/braincomms/fcab199)

**Supplementary Material 1 Neuroimaging data acquisition and pre-processing within the IMAP+ primary cohort.**

***MRI data acquisition and pre-processing***

A high-resolution T1-weighted anatomical image was acquired on a 3T Philips Achieva MRI scanner, using a 3D fast-field echo sequence (3D-T1-FFE sagittal; repetition time = 20 ms; echo time = 4.6 ms; flip angle = 10°; 180 slices with no gap, slice thickness = 1 mm, field of view = 256×256 mm²; in-plane resolution = 1×1 mm²). T1-weighted MRI were iteratively segmented and spatially normalized to the Montreal Neurological Institute (MNI) space. Then, the normalized grey matter segments were modulated to correct for non-linear warping effects and the resultant images were smoothed using a 8 mm full-width half-maximum (FWHM) Gaussian kernel (Chételat *et al.*, 2008; Villain *et al.*, 2008; La Joie *et al.*, 2012) using the voxel-based morphometry toolbox (VBM12) implemented in SPM12 software (Statistical Parametric Mapping, [www.fil.ion.ucl.ac.uk/spm](http://www.fil.ion.ucl.ac.uk/spm)). All resultant MRI images were finally masked to exclude non-grey matter voxels as well as the cerebellum from the analyses.

***PET data acquisition and pre-processing***

Both 18F-fluorodeoxyglucose (FDG) and florbetapir-PET scans were acquired in two separate sessions, with a Discovery RX VCT 64 PET-CT scanner (General Electric Healthcare); with a resolution of 3.76 x 3.76 x 4.9mm3 (field of view = 157 mm). Forty-seven planes were obtained with a voxel size of 1.95 x 1.95 x 3.2mm3. A transmission scan was performed for attenuation correction before the PET acquisition. For 18F-FDG-PET, the participants were fasted for at least 6 hours before scanning. After a 30 min resting period in a quiet and dark environment, 180 MBq of 18F-FDG was intravenously injected as a bolus. A 10 min PET acquisition scan began 50 min after injection. For florbetapir-PET, each participant underwent a 20 min PET scan, beginning 50 min after the intravenous injections of ~4 MBq/kg of florbetapir.

PET data were coregistered onto their corresponding MRI, and normalized using the deformation parameters defined from the MRI procedure. Resultant images were quantitatively normalized using the cerebellar grey matter as the reference region. As PET and T1-weighted anatomical images did not have the same original spatial resolution, a differential Gaussian kernel smoothing was applied to obtain an equivalent data effective smoothing of 10 mm FWHM (Chételat *et al.*, 2008; Villain *et al.*, 2008; La Joie *et al.*, 2012; Bejanin *et al.*, 2019). All resultant PET images were finally masked to exclude non-grey matter voxels as well as the cerebellum from the analyses.

The global neocortical standardized uptake value ratio (SUVr) value was also obtained in each participant from the Florbetapir-PET non-corrected for partial volume effects (NoPVC) SUVr images using a neocortex mask (including all regions but the cerebellum, occipital and sensori motor cortices, hippocampus, amygdala and subcortical grey nuclei) (La Joie *et al.*, 2013). The SUVr was used to classify participants as florbetapir positive or negative, using a threshold derived from an independent group of 41 young individuals from the IMAP project (16 females; age = 28.40 ± 6.06 years) (Besson *et al.*, 2015; Perrotin *et al.*, 2017). The positivity threshold was defined by the mean + 2 SD of 41 healthy young controls aged 21 to 39 years old (supposedly devoid of amyloid deposition), corresponding to a Florbetapir SUVr of 1.24. Participants with values above this threshold were considered as amyloid-positive and those below this threshold as amyloid-negative.

***References***

Bejanin A, La Joie R, Landeau B, Belliard S, de La Sayette V, Eustache F, et al. Distinct Interplay Between Atrophy and Hypometabolism in Alzheimer’s Versus Semantic Dementia. Cereb Cortex 2019; 29: 1889–99.

Besson FL, Joie RL, Doeuvre L, Gaubert M, Mézenge F, Egret S, et al. Cognitive and Brain Profiles Associated with Current Neuroimaging Biomarkers of Preclinical Alzheimer’s Disease. J Neurosci 2015; 35: 10402–11.

Chételat G, Desgranges B, Landeau B, Mézenge F, Poline JB, de la Sayette V, et al. Direct voxel-based comparison between grey matter hypometabolism and atrophy in Alzheimer’s disease. Brain 2008; 131: 60–71.

La Joie R, Perrotin A, Barré L, Hommet C, Mézenge F, Ibazizene M, et al. Region-Specific Hierarchy between Atrophy, Hypometabolism, and β-Amyloid (Aβ) Load in Alzheimer’s Disease Dementia. J Neurosci 2012; 32: 16265–73.

La Joie R, Perrotin A, de La Sayette V, Egret S, Doeuvre L, Belliard S, et al. Hippocampal subfield volumetry in mild cognitive impairment, Alzheimer’s disease and semantic dementia. NeuroImage: Clinical 2013; 3: 155–62.

Perrotin A, La Joie R, de La Sayette V, Barré L, Mézenge F, Mutlu J, et al. Subjective cognitive decline in cognitively normal elders from the community or from a memory clinic: Differential affective and imaging correlates. Alzheimer’s & Dementia 2017; 13: 550–60.

Villain N, Desgranges B, Viader F, Sayette V de la, Mézenge F, Landeau B, et al. Relationships between Hippocampal Atrophy, White Matter Disruption, and Gray Matter Hypometabolism in Alzheimer’s Disease. J Neurosci 2008; 28: 6174–81.

**Supplementary Table 1 Sample sizes according to neuroimaging analyses in both independent cohorts.**

| **N** | **Controls** | **SCD** | **MCI** | **Dementia** | **Entire Sample** |
| --- | --- | --- | --- | --- | --- |
| **IMAP+ primary cohort** | 67  (17) | 36  (9) | 60  (34) | 37  (29) | 200  (89) |
| Correlation MRI ~ SMD | 64  (17) | 30  (9) | 50  (30) | 36  (28) | 180  (84) |
| Correlation NoPVC FDG-PET ~ SMD | 59  (17) | 29  (9) | 49  (30) | 33  (28) | 170  (84) |
| Interaction MRI ~ SMD * MMSE |  |  |  |  | 178  (84) |
| Interaction NoPVC FDG-PET ~ SMD * MMSE |  |  |  |  | 168  (84) |
| Interaction MRI ~ SMD * wESR |  |  |  |  | 172  (77) |
| Interaction NoPVC FDG-PET ~ SMD * wESR |  |  |  |  | 162  (77) |
| **ADNI replication cohort** | 157  (89) | 84  (49) | 369  (257) | 121  (108) | 731  (503) |
| Correlation MRI ~ SMD | 157  (89) | 84  (49) | 369  (257) | 121  (108) | 731  (503) |
| Correlation NoPVC FDG-PET ~ SMD | 157  (89) | 84  (49) | 369  (257) | 121  (108) | 731  (503) |
| Interaction MRI ~ SMD * MMSE |  |  |  |  | 731  (503) |
| Interaction NoPVC FDG-PET ~ SMD * MMSE |  |  |  |  | 731  (503) |
| Interaction MRI ~ SMD * wRAVLT |  |  |  |  | 730  (502) |
| Interaction NoPVC FDG-PET ~ SMD * wRAVLT |  |  |  |  | 730  (502) |

Values expressed as sample size (sample size when only including the amyloid-positive participants).

wESR = w-score of Encoding, Storage and Retrieval, 16 words list delayed recognition subscores; wRAVLT = w-score of Rey Auditory Verbal Learning Test, 15 words list subscores (five first trials).

**Supplementary Table 2 Determination of minimal cluster sizes for neuroimaging analyses.**

| **Statistical design – Multiple regressions** | **Minimal cluster sizes (k voxels)** | | |
| --- | --- | --- | --- |
|  | **MRI** | **FDG-PET** | |
|  |  | NoPVC | PVC |
| **IMAP+ primary cohort** |  |  |  |
| Correlation Neuroimaging ~ SMD | 240  (249) | 1859  (1482) | 1610 |
| Interaction Neuroimaging ~ SMD * Global cognitive performances (MMSE) | 205  (188) | 2047  (1810) | 1708 |
| Interaction Neuroimaging ~ SMD * Memory performances (wESR) | 206  (205) | 2197  (2239) | 1849 |
| **ADNI replication cohort** |  |  |  |
| Correlation Neuroimaging ~ SMD | 228  (218) | 1931  (1897) | 1888 |
| Interaction Neuroimaging ~ SMD * Global cognitive performances (MMSE) | 222  (215) | 2005  (2002) | 1787 |
| Interaction Neuroimaging ~ SMD * Memory performances (wRAVLT) | 216  (222) | 1825  (2064) | 1694 |

Minimal cluster sizes (k) were determined for each statistical design at the p_uncorrected_ < 0.005 level by Monte-Carlo simulation using the Clustersim program, in order to achieve a corrected statistical significance of p < 0.05. Values expressed as total participants (amyloid positive participants).

wESR = w-score of Encoding, Storage and Retrieval, 16 words list delayed recognition subscores; wRAVLT = w-score of Rey Auditory Verbal Learning Test, 15 words list subscores (five first trials).

**Supplementary Table 3 Relationships between education and the SMD or delta score within each clinical group.**

|  | **Controls** | **SCD** | **MCI** | **Dementia** |
| --- | --- | --- | --- | --- |
| **IMAP+ primary cohort** | | | | |
| Education – SMD | r= 0.18  p= 0.15 | r= 0.14  p=0.35 | r= 0.05  p= 0.74 | r= 0.05  p=0.79 |
| Education - Delta | r= 0.16  p= 0.20 | r= 0.15  p= 0.30 | r= -0.01  p= 0.94 | r= -0.16  p= 0.43 |
| **ADNI replication cohort** | | | | |
| Education – SMD | r= -0.005  p= 0.95 | r= -0.08  p= 0.49 | r= -0.05  p= 0.33 | r= 0.08  p=0.37 |
| Education - Delta | r= 0.11  p= 0.18 | r= 0.11  p= 0.35 | r= 0.04  p= 0.41 | r= 0.17  p=0.06 |

**Supplementary Fig. 1 Negative interactive effect of the level of memory impairment (ESR/RAVLT w-score according to the cohort) on the voxelwise relationships between the SMD score and neurodegeneration.** Brain representations (left panel) show the results of the voxelwise negative interactions between the SMD and ESR/RAVLT w-scores on either glucose metabolism (green) or grey matter volume (blue) thresholded at p<0.005 combined with a cluster-level corrected for multiple comparisons; and the graphs (right panel) illustrate the regression in the corresponding brain areas for each ESR/RAVLT tertile. All analyses were adjusted for age, sex and education. **A.** Data for the IMAP+ primary cohort. **B.** Data for the ADNI replication cohort. NS = Not significant, wESR = w-score of Encoding, Storage and Retrieval, 16-words list delayed recognition subscores; wRAVLT = w-score of Rey Auditory Verbal Learning Test, 15-words list subscores (five first trials).


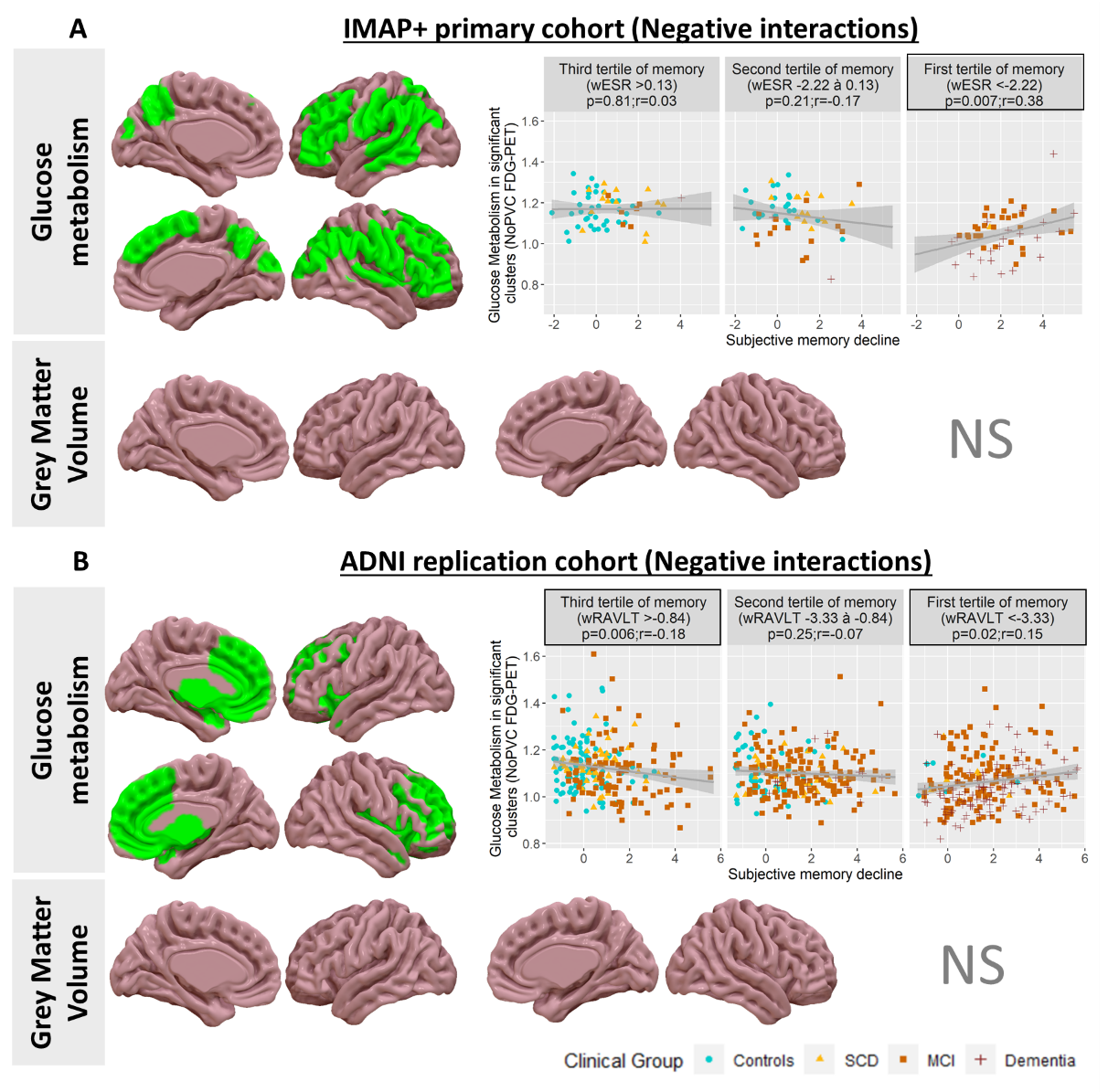


**Supplementary Fig. 2 Voxelwise relationships between the SMD score and glucose metabolism when using PVC FDG-PET images within each clinical group.** Brain representations show the results of the voxelwise correlations between the SMD score and glucose metabolism with PVC FDG-PET thresholded at p<0.005 combined with a cluster-level corrected for multiple comparisons (green; except one thresholded at p<0.005 and k>100 voxels in yellow); and the graphs on their right side illustrate the corresponding regressions. All analyses were adjusted for age, sex and education. **A.** Data for the IMAP+ primary cohort. **B.** Data for the ADNI replication cohort. NS = Not significant.


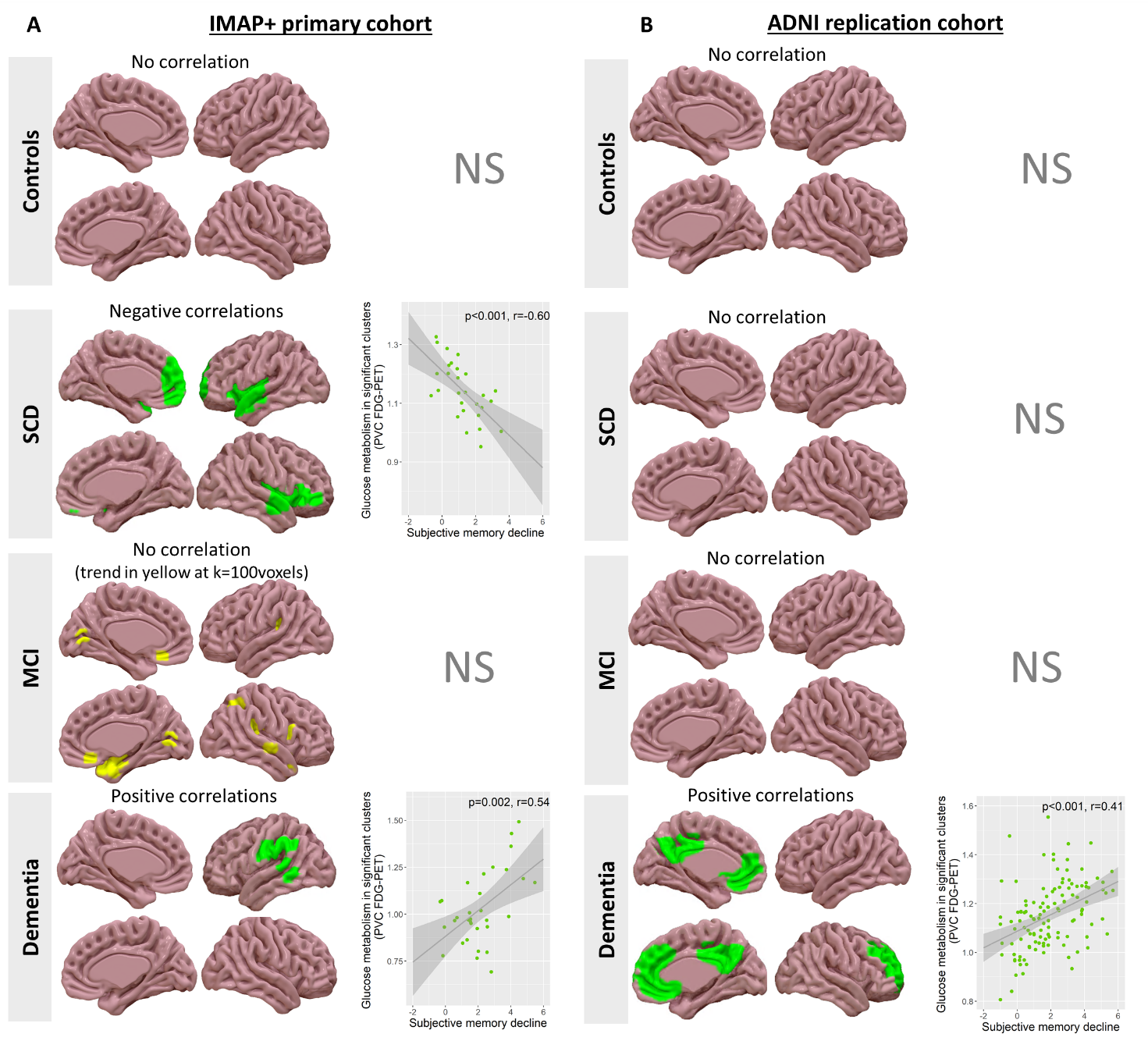


**Supplementary Fig. 3 Negative interactive effect of the level of cognitive/memory impairment on the voxelwise relationships between the SMD score and glucose metabolism when using PVC FDG-PET imaging**. Brain representations (left panel) show the result of the voxelwise negative interactions between the SMD and the global cognitive or memory scores (A1/B1. MMSE, A2. wESR or B2 wRAVLT), on glucose metabolism measured with PVC FDG-PET (green) thresholded at p<0.005 combined with a cluster-level corrected for multiple comparisons; and the graphs (right panel) illustrate the regression in the corresponding brain areas for each tertile of MMSE or ESR/RAVLT score. All analyses were adjusted for age, sex and education. **A.** Data for the IMAP+ primary cohort. **B.** Data for the ADNI replication cohort. wESR = w-score of Encoding, Storage and Retrieval, 16 words list delayed recognition subscores; wRAVLT = w-score of Rey Auditory Verbal Learning Test, 15 words list subscores (five first trials).


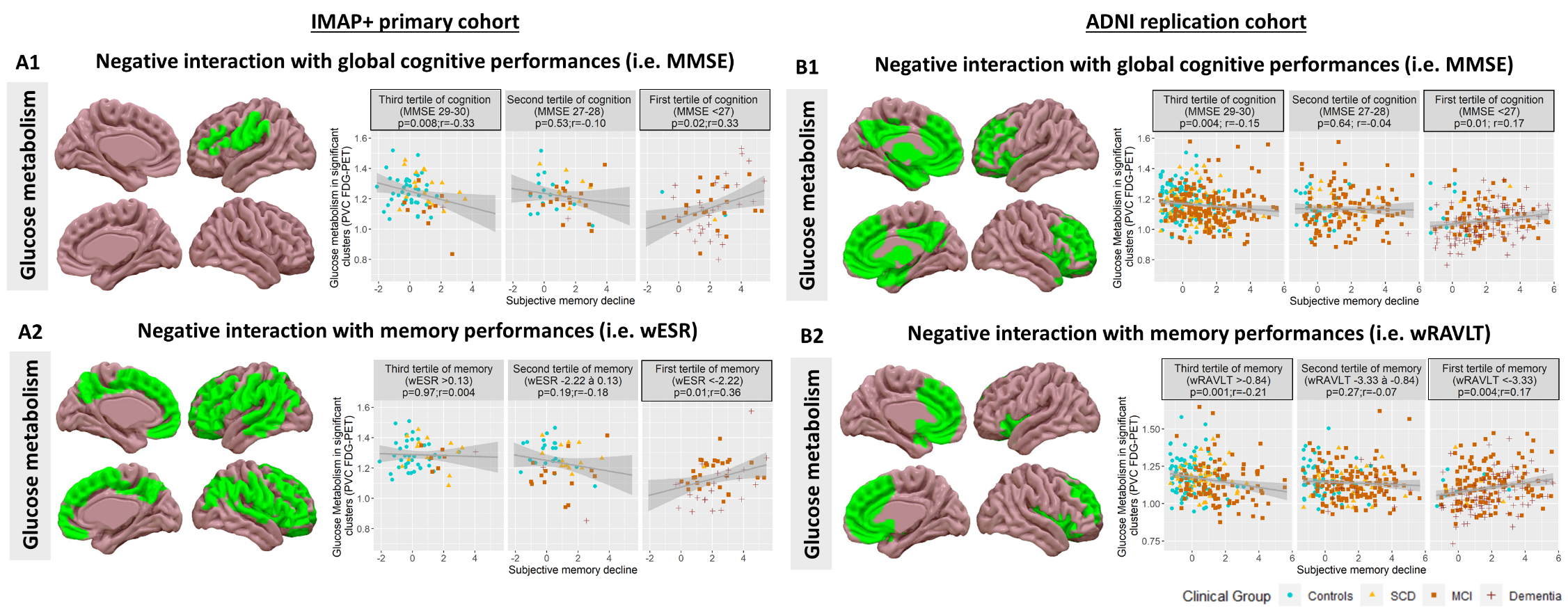


**Supplementary Fig. 4 Voxelwise relationships between the SMD score and neurodegeneration within each clinical group when only including the amyloid-positive participants.** Brain representations show the results of the voxelwise correlations between the SMD score and either glucose metabolism (green) or grey matter volume (blue) thresholded at p<0.005 and k>100 voxels (and when available combined with a cluster-level corrected for multiple comparisons in darkgreen and darkblue respectively); and the graphs on their right side illustrate the corresponding regressions (at k>100 voxels). All analyses were adjusted for age, sex and education. **A.** Data for the IMAP+ primary cohort. **B.** Data for the ADNI replication cohort. NS = Not significant.


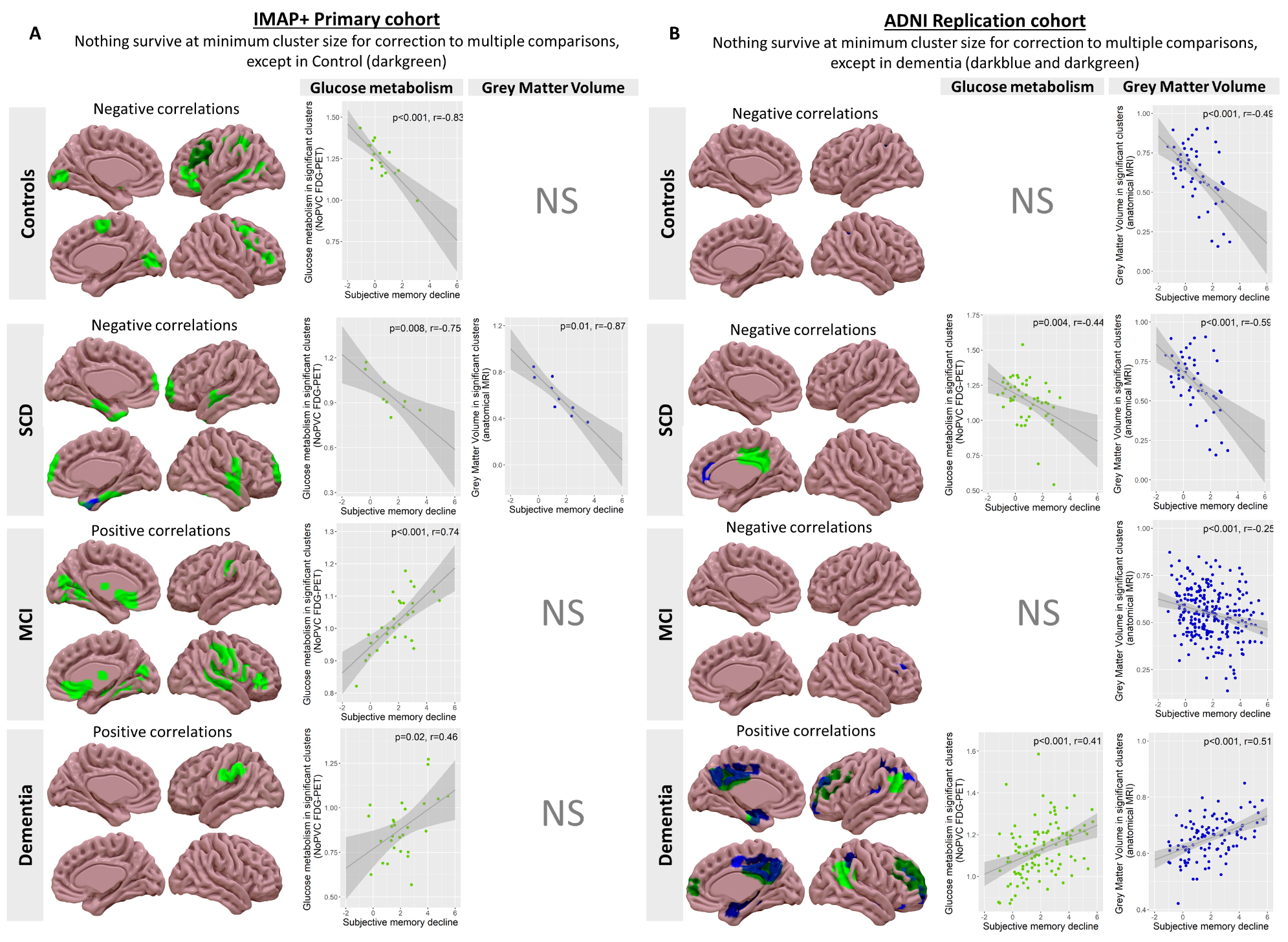


**Supplementary Fig. 5 Negative interactive effect of the level of cognitive/memory impairment on the voxelwise relationships between the SMD score and neurodegeneration when only including amyloid-positive participants.** Brain representations (left panel) show the result of the voxelwise interactions between the SMD and the global cognitive or memory scores (A1/B1. MMSE, A2. wESR or B2 wRAVLT), on either glucose metabolism (green) and grey matter volume (blue) thresholded at p<0.005 and k>100 voxels (and when available combined with a cluster-level corrected for multiple comparisons in darkgreen and darkblue respectively); and the graphs (right panel) illustrate the regression in the corresponding brain areas for each tertile of MMSE or ESR/RAVLT score. All analyses were adjusted for age, sex and education. **A.** Data for the IMAP+ primary cohort. **B.** Data for the ADNI replication cohort. NS = Not significant, wESR = w-score of Encoding, Storage and Retrieval, 16 words list delayed recognition subscores; wRAVLT = w-score of Rey Auditory Verbal Learning Test, 15 words list subscores (five first trials).


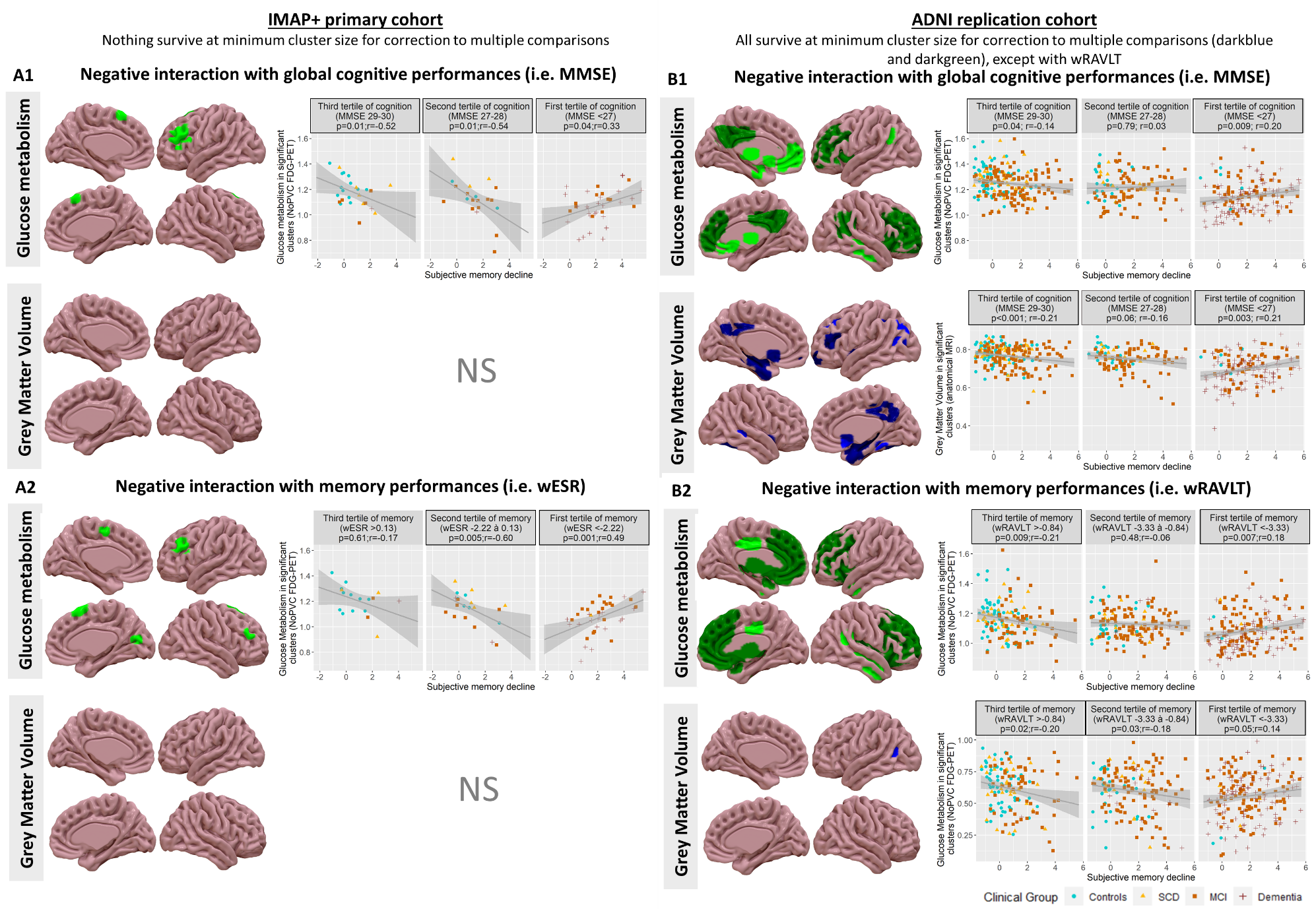


**Supplementary Fig. 6 Voxelwise relationships between the subjective cognitive (instead of memory) decline score and neurodegeneration within each clinical group.** Brain representations show the results of the voxelwise correlations between the subjective cognitive decline score and either glucose metabolism (green) or grey matter volume (blue) thresholded at p<0.005 combined with a cluster-level corrected for multiple comparisons; and the graphs on their right side illustrate the corresponding regressions. All analyses were adjusted for age, sex and education. **A.** Data for the IMAP+ primary cohort. **B.** Data for the ADNI replication cohort. NS = Not significant.


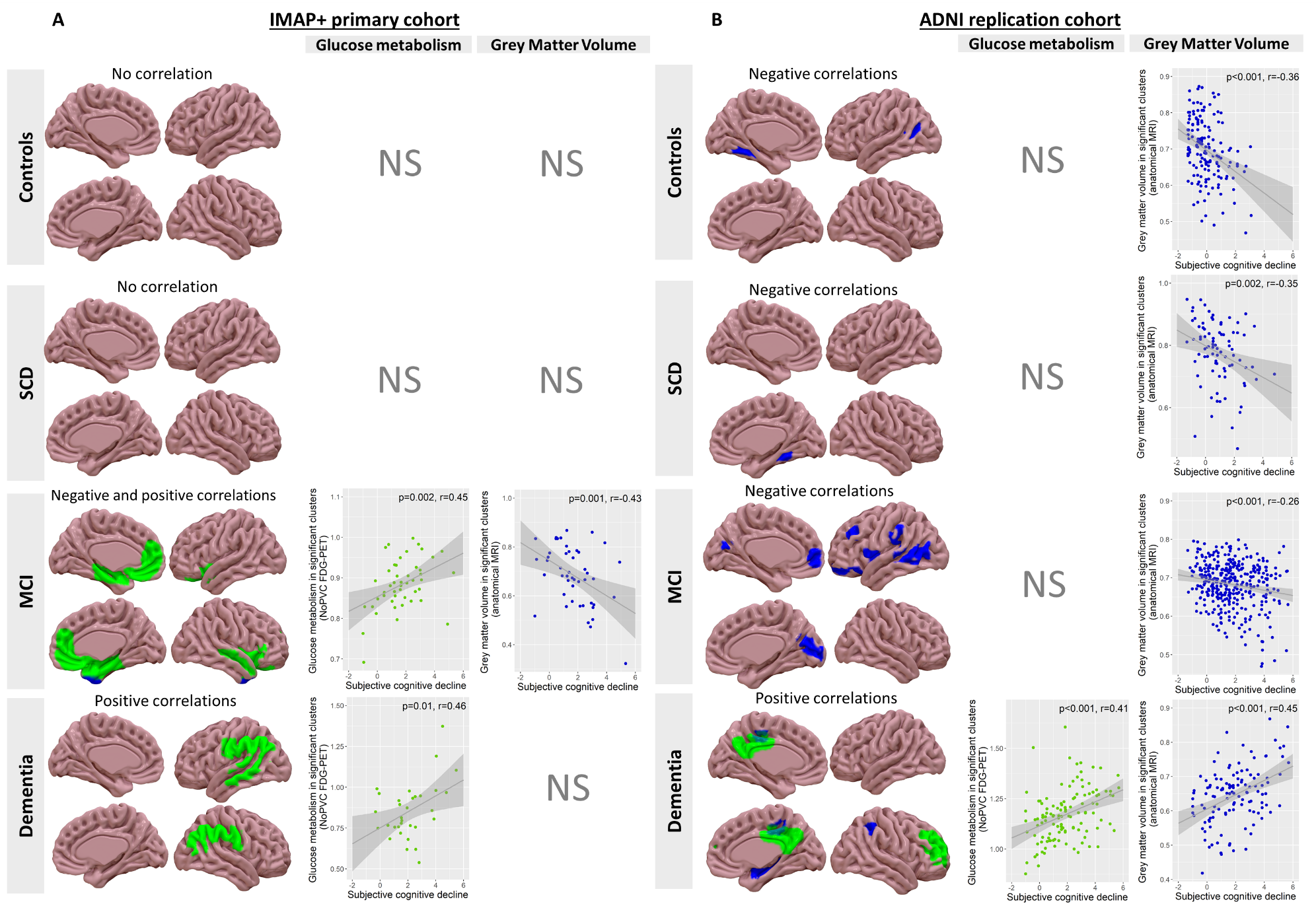


**Supplementary Fig. 7 Negative interactive effect of the level of cognitive/memory impairment on the voxelwise relationships between the subjective cognitive decline score (instead of memory) and neurodegeneration.** Brain representations (left panel) show the result of the voxelwise interactions between the subjective and objective cognitive scores (A1/B1. MMSE, A2. wESR or B2. wRAVLT), on either glucose metabolism (green) and grey matter volume (blue) thresholded at p<0.005 and cluster-level corrected for multiple comparisons; and the graphs (right panel) illustrate the regression in the corresponding brain areas for each tertile of MMSE or ESR/RAVLT score. All analyses were adjusted for age, sex and education. **A.** Data for the IMAP+ primary cohort. **B.** Data for the ADNI replication cohort. NS = Not significant, wESR = w-score of Encoding, Storage and Retrieval, 16 words list delayed recognition subscores; wRAVLT = w-score of Rey Auditory Verbal Learning Test, 15 words list subscores (five first trials).


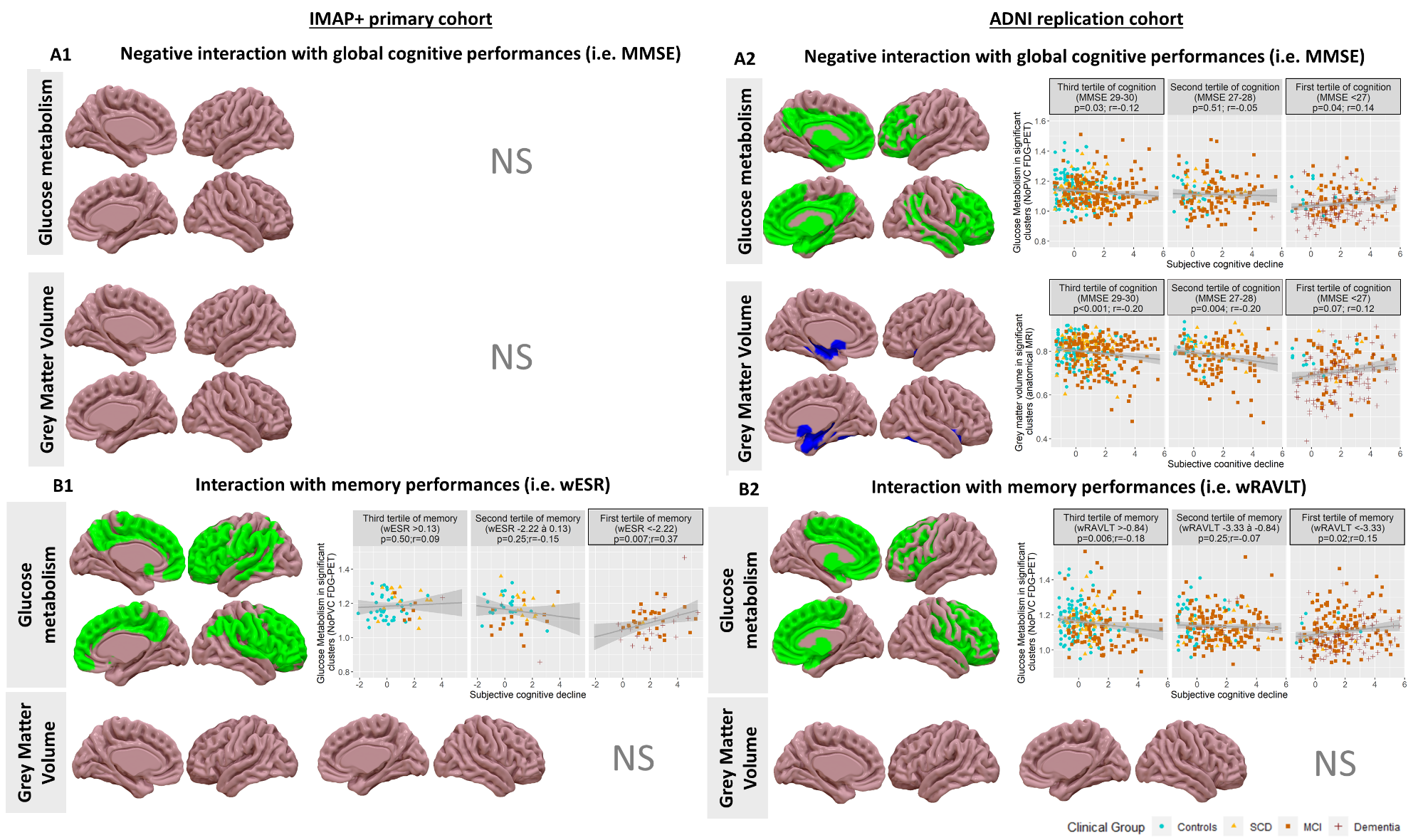

Supplement: fcab199_Supplementary_Data [file fcab199_supplementary_data.docx]
